# Role of metal ions in the cognitive decline of Down syndrome

**DOI:** 10.3389/fnagi.2014.00136

**Published:** 2014-06-23

**Authors:** Nakisa Malakooti, Melanie A. Pritchard, Paul A. Adlard, David I. Finkelstein

**Affiliations:** ^1^Oxidation Biology Unit, Florey Institute of Neuroscience and Mental HealthParkville, VIC, Australia; ^2^Department of Biochemistry, Monash UniversityClayton, VIC, Australia

**Keywords:** Down syndrome, Alzheimer disease, iron, copper, zinc, calcium

## Abstract

Down syndrome (DS), caused by trisomy of whole or part of chromosome 21 is the most common mental impairment. All people with DS suffer from cognitive decline and develop Alzheimer’s disease (AD) by the age of 40. The appearance of enlarged early endosomes, followed by Amyloid βpeptide deposition, the appearance of tau-containing neurofibrillary tangles and basal forebrain cholinergic neuron (BFCN) degeneration are the neuropathological characteristics of this disease. In this review we will examine the role of metal ion dyshomeostasis and the genes which may be involved in these processes, and relate these back to the manifestation of age-dependent cognitive decline in DS.

## Introduction

This review will discuss the role of intrinsic metals in cognitive decline in people with Down syndrome (DS). DS, caused by trisomy of whole or part of human chromosome 21 is the most common mental impairment with an incidence of about 1 in 700 live births (Epstein, 1995). Characteristics of DS individuals have been described such as stunted growth, mental impairment, congenital disorders of multi-organ systems such as the heart, haematological, musculoskeletal, thyroid, gastrointestinal, dental, nervous and immune systems, and having a higher incidence of diabetes and age-associated diabetes (Burch and Milunsky, 1969; Peiris et al., 2012). Life expectancy of people with DS has increased from 12 years in the 1940s to 60 years at the present time, largely due to enhanced medical and social care (Contestabile et al., 2010). Because of the increased longevity, it has become evident that by the age of 40 (Mann et al., 1990), all individuals with DS have the cognitive decline and the neuropathology seen in Alzheimer’s disease (AD).

AD is the most common form of dementia. It was first described by a German psychiatrist and neuropathologist named Alois Alzheimer in 1906 (Alzheimer et al., 1995). One of the earlier symptoms is short term memory loss. As the disease progresses long-term memory loss, confusion and mood swing occur. Sporadic AD and DS share similar neuropathological features (Mann, 1988), these include the enlargement of early endosomes in certain neurons, deposition of Aβ plaques, the presence of tau-containing neurofibrillary tangles and the degeneration of basal forebrain cholinergic neuron (BFCN). The pattern of pathology is similar but the symptoms appear earlier in individuals with DS (Ikeda et al., 1989). In DS, enlarged endosomes in neurons are seen as early as 28 weeks of gestation (Cataldo et al., 2000). The presence of enlarged endosomes precedes Aβ peptide deposition which appears at around 12 years of age in the form of diffuse plaques, followed by mature Aβ plaques when the individuals are in their 30’s (Lemere et al., 1996). In a review of the literature, it was reported that most studies have found that tau-containing neurofibrillary tangles occur in the BFCN along with neuronal degeneration, gliosis and dementia at ages of between 35 to 45 years (Wilcock and Griffin, 2013). An immunohistochemical study of the brain tissues from post-mortem DS individuals showed that in earlier years of life (37–38) brain neuron loss was present and this progressed with age (over 50s) with abundant, mature amyloid deposits. Taken together, these observations suggest that in DS there is an accelerated progression occurring through the different stages of AD-like neuropathology.

There are 127 known genes, 98 predicted genes and 59 pseudo genes located on chromosome 21 (Hattori et al., 2000). It is putatively thought that having an extra copy of one or more of these genes could be responsible for the manifestation of AD-like neuropathology and cognitive decline in DS. Some genes of interest are: Amyloid precursor protein (*APP*), Down syndrome candidate region 1 (*DSCR1*), Intersectin 1(*ITSN1*), Superoxide dismutase 1(*SOD1*), Beta-site APP-cleaving enzyme 2 (βsecretase or *BACE 2*), S100 calcium binding protein β(*S100*β).

In the next section, how these genes may be involved in cognitive decline of DS will be discussed.

## Genes potentially involved in cognitive decline of DS

### Amyloid precursor protein (APP)

Studies have suggested that over-expression of the APP is the key element for the manifestation of AD in DS (Schupf and Sergievsky, 2002). A case study where an individual had partial trisomy 21 that did not include an extra copy of the *APP* gene confirmed this concept (Prasher et al., 1998). This individual showed some characteristics of DS but did not suffer from AD and the associated cognitive decline.

Amyloid peptides (Aβ 1–40/42) are cleavage products of the APP. These form β-sheet rich aggregates and soluble oligomers. The cleavage products are generated by the proteolytic enzymes βsecretase and γsecretase. There is also an alternative pathway where α-secretase cleaves APP. The products of this pathway do not include Aβ peptides and aggregates do not form (Vassar et al., 1999). The gene encoding βsecretase 2 is located on chromosome 21, therefore, an extra copy would potentially contribute to the production of higher levels of toxic Aβ, hence formation of amyloid plaques early in DS.

However, this extra dosage of APP and BACE may not be required to produce the AD-like phenotype in DS. This idea can be derived from observations from an individual who had an over-expression of APP, but exhibited normal levels of BACE (Cheon et al., 2008). In a recent study, polymorphisms in BACE2 were shown to be a major factor for the age of onset of AD in the DS population (Mok et al., 2014) suggesting a significant role for BACE2 in the development of AD in DS.

In addition, it was shown that the amyloid truncated Aβ peptides (Aβ 9–42 and Aβ 17–42) form ion channels by formation of a β-barrel secondary structure (βstrand-turn- βstrand) that alters calcium (Ca^2+^) homeostasis providing additional avenues for the manifestation of AD (Jang et al., 2010). An alternative mechanism may be that N-terminal truncated amyloids are neurotoxic because they form fibrils that aggregate more readily (Pike et al., 1995). These studies suggest that it is not only Aβ but smaller truncated amyloid peptides which are also involved in the development of AD.

AD research has focused heavily on APP. However studies have suggested that APP may not be the only factor involved in the manifestation of AD. This was demonstrated in mice where the APP protein was over-expressed in a mouse model of DS (Ts65Dn). No change in the endosomal phenotype was observed indicating other factors are necessary for this alteration (Cataldo et al., 2003). As mentioned above, there are other genes that could contribute to the development of AD-like neuropathology that will be discussed later in this review. Although APP is necessary to develop the disease, it appears that it alone is not sufficient to cause it. This is why it is interesting to study DS as it narrows the search done to trisomy genes.

Importantly, amyloid plaques are formed as the result of inappropriate interaction between biometals (iron, copper and zinc) and beta-amyloid. Indeed, increased levels of zinc promote Aβ precipitation (Bush et al., 1994). There is also increased levels of iron in AD patients (Gerlach et al., 1994). These observed increases in the levels of metals in AD give rise to the questions of why, how and when these increases occur? This so-called metal theory of AD and DS will be discussed later in this review.

### Down syndrome candidate region 1

Studies showed that DSCR1 mRNA expression was increased in post-mortem brains of AD patients compared with aged-matched non-AD controls (Ermak et al., 2001). DSCR1 is a regulator of calcineurin and in some cases has been shown to inhibit calcineurin signaling pathways (Fuentes et al., 2000). Over-expression of DSCR1 contributes to the formation of neurofibrillary tangles and Aβ deposition (Ermak et al., 2011). As discussed earlier, formation of neurofibrillary tangles is the first step of memory loss. Hence, having an extra copy of DSCR1 in DS could contribute to earlier formation of neurofibrillary tangles and premature cognitive decline. The formation of tangles could be facilitated by the enhanced ability of over-activate calcineurin to dephosphorylate tau but the mechanism has not been fully elucidated (Ermak et al., 2001; Ma et al., 2004).

### Intersectin 1

Another potential gene of interest on chromosome 21 is *ITSN1*. This gene appears to be involved in metal homeostasis since it is involved in receptor mediated endocytosis and one of its cargos is the transferrin/transferrin receptor complex required for iron transport into the cell. Transferrin (and its iron cargo) is transferred from the blood into cells by the formation of a vesicle (clathrin-coated pit) that internalizes the complex. First the recruitment of adaptor protein complex 2 (AP-2) to the plasma membrane occurs, and then clathrin is recruited to the cytoplasm where AP-2 has been recruited. The plasma membrane forms a vesicle by budding inward. The receptors and bound ligand (transferrin) are thus taken up by the cell. ITSN1 is involved in the recruitment of AP-2 and the formation of clathrin-coated pits. Having an extra copy of the *ITSN1* gene in DS could alter receptor trafficking and consequently iron transport.

## Metals

### The metal theory

As discussed above, excess APP and/or its cleavage products is necessary for the development of AD but, it is not sufficient to cause it. In addition, clinical trials targeting Aβ (by anti- Aβ antibodies) have failed to treat AD (Greenberg et al., 2003). Bush et al. (on the basis of the studies done by them and others) have proposed that a number of proteins involved in neurodegeneration (Aβ, AβPP, tau, presenilin, and β-secretase1) fail in their ability to regulate metals in the AD brains (Finefrock et al., 2003; Bonda et al., 2011; Bush, 2013), and consequently these proteins are overwhelmed by the increased levels of these metals. This leads to accumulation of extracellular zinc and copper in amyloid, and an accumulation of intracellular iron in neurons (Bush, 2013).

#### Iron

Iron is essential for normal neurological function. It is required for the synthesis of myelin and neurotransmitters (Piñero and Connor, 2000; Bush, 2013). It is known that free iron accumulates in the brains of AD patients, and that iron transport and storage are disrupted (Sayre et al., 2000). Another study showed that iron stimulates the production of hydroxyl radicals from hydrogen peroxide, and hence exacerbates oxidative stress. Iron was increased in all areas of the AD brains compared with controls and transferrin was increased in the frontal cortex of AD brains. The transferrin/iron ratio which is indicative of iron mobilization was reduced in AD brains, indicating a disturbance of iron metabolism in the AD population (Loeffler et al., 1995).

Iron transport into the brain is accomplished by Transferrin (Tf). Tf is a glycoprotein that carries iron in the blood and regulates iron homeostasis. As mentioned above, ITSN1 is required for iron internalization. It has been reported that there is an increase of Tf in brains of DS individuals compared with controls (Leveugle et al., 1994). An extra copy of *ITSN1* could be the reason for this elevated level of Tf in DS brains, and this level increases with age in DS. The increased level of Tf is also evident in the amniotic fluid obtained from women carrying DS foetuses (Perluigi et al., 2011), suggesting iron-mediated damage starts during pregnancy.

Ferritin, the iron storage protein is available in the cytosol to bind the iron that has been endocytosed into the cell. Excess iron can be transported out of the cell by ferroportin (Fpn). For this to occur, a ferroxidase enzyme is required. In the glia, ceruloplasmin is the known ferroxidase (Klomp et al., 1996) but ceruloplasmin is not produced by neurons. Instead, APP has been implicated in the process of iron export in neuronal cells. This occurs through the stabilization of Fpn (Duce et al., 2010). Interestingly, excess dietry iron fed to APP-knock-out mice induced iron accumulation and damaged the neocortex (Duce et al., 2010).

Iron regulatory protein (IRP) is a cytoplasmic protein that regulates iron uptake, storage and usage in response to the concentration of cellular iron. The level of iron regulatory protein (IRP-2) in the whole brain was the same in both AD patients and controls. However IRP-2 is co-localized to the specific areas with AD neuropathology (Farrar et al., 1990; Smith et al., 1998), suggesting the regulation of iron uptake, storage and usage changes in specific parts of AD brains which leads to the development of the AD neuropathology. The role of IRP-2 and indeed iron in the patho-biology of AD has been well established, whereas in DS this needs to be investigated.

Tau protein stabilizes microtubules which are involved in cellular transport. In AD, excess tau phosphorylation causes formation of tau clumps (neurofibrillary tangles). This leads to a break down of nutrient transport and death of the neurons which is believed to be the first step of memory loss. The appearance of tau-containing neurofibrillary tangles is one of the later neuropathological hallmarks in AD brains. Tau can bind to iron and this could contribute to tau aggregation (Adlard and Bush, 2006). Tau protein is needed for iron-export as it was shown that iron accumulated in tau-knock-out mice (Lei et al., 2012). Therefore, excess iron levels in the brain could contribute to memory loss as the consequence of an increased level of tau aggregation.

#### Copper

The brain is enriched with copper. Copper has been localized in mitochondria, synaptosomes and myelin (Nalbandyan, 1983). In a normal nervous system, copper plays a role in regulating neuronal excitability (Kardos et al., 1989), myelination, iron metabolism and the function of copper containing enzymes such as superoxide dismutase (SOD), dopamine-β monooxygenase and tyrosinase (Nalbandyan, 1983). As mentioned above, the *SOD1* gene is located on chromosome 21. An extra copy of *SOD1* in DS might contribute to a dysfunctional SOD1 enzyme. SOD is a copper and zinc binding enzyme which is responsible for destroying free superoxide radicals in the body.

Studies have also shown that APP knockout mice have higher levels of copper in brain and liver (White et al., 1999) while the APP-overexpressing transgenic mice have reduced levels (Maynard et al., 2002) indicating that APP acts as a major regulator of neuronal copper homeostasis. On the other hand, a recent study showed that copper promotes APP trafficking through the secretory pathway (Acevedo et al., 2014). Collectively, these studies suggest there is a two way relationship between copper and APP.

It is known that copper binding to APP reduces the production of Aβ **in vitro** (Barnham et al., 2003). As DS individuals have an extra copy of the *APP* gene, it may follow that they would have reduced levels of copper in their brains and hence increased production of Aβ leading to the formation of amyloid plaques. This has yet to be investigated.

It has also been shown that in AD brains, there is an overall deficiency of copper (Cater et al., 2008) and an increased level of extracellular copper in amyloid (Bush, 2013) suggesting a change in the homeostasis of copper in AD. Excess cellular copper can compromise cell viability by acting as a pro-oxidant and generating toxic reactive oxygen species via Fenton-type reactions, involving copper ions and hydrogen peroxide. Therefore, cellular copper homeostasis must be tightly regulated (Huang et al., 1999) to maintain healthy brain function.

#### Zinc

Zinc is essential for the function of numerous enzymes and transcription factors. In the normal brain, zinc is bound to membrane-bound metalloproteins, or loosely bound within the cytoplasm to proteins and enzymes as well as being in synaptic vesicles that are enriched with zinc (exceeding 1 mmol/L in concentration) (Frederickson et al., 2000). During synaptic transmission, high concentrations of zinc are released into the synapse from mossy fibers (Sensi et al., 2011). Zinc regulates ion channels and transmitter receptors such as α-Amino-3-hydroxy-5-methyl-4-isoxazolepropionic acid (AMPA) and N-methyl-D-aspartate (NMDA) receptors which are implicated in synaptic plasticity and memory consolidation (Sindreu and Storm, 2011). Therefore, any change in homeostasis of zinc can have an impact on learning and memory.

Within the ectodomain 2 (E2) of APP, a metal binding site has been identified which binds competitively to zinc or copper (Dahms et al., 2012). Both zinc and copper can interact with Aβ to form aggregates (Adlard and Bush, 2006). An increase in level of extracellular zinc in amyloid of AD has been observed (Bush, 2013). Earlier studies demonstrated that a small increase in brain zinc concentration (>3 micromolar) increased the adhesiveness of Aβ (Bush et al., 1994) and changed Aβ metabolism. Intriguingly, the area of the brain with the highest level of zinc, the cerebral cortex (Frederickson et al., 1983), exhibits the most severe pathological lesions of AD (Hyman et al., 1986).

Zinc transporters (ZnT) are also crucial to maintain memory and cognitive function. There are 10 ZnT known to date. Zinc uptake into synaptic vesicles needs zinc-transporter-3 (Cole et al., 1999). It has been demonstrated that zinc-transporter-3 knock-out mice develop amyloid pathology characteristic of AD (Adlard et al., 2010) supporting a role for the zinc transporter in the manifestation of AD. Another study showed that zinc-transporter-6, which is located in the Golgi apparatus, is involved in the accumulation of zinc (Lyubartseva et al., 2010). The role of ZnT is yet to be investigated in DS.

#### Calcium (Ca^2+^)

Calcium plays an important role in the central nervous system. It acts as a cofactor, second messenger and signaling molecule, and a coenzyme when part of a protein. It is also part of excitatory function of neuronal cells, being involved in voltage-gated calcium ion channels. It has been shown that voltage-gated calcium ion channel activity in a mouse model of DS (trisomy 16 mouse; Ts16) was significantly higher than the wild type (Galdzicki et al., 1998). This increased activity could potentially lead to altering calcium homeostasis in the brains of DS individuals.

Human chromosome 21 has two genes of interest that modulate calcium; the *S100*β gene stimulates calcium influx (Mattson et al., 1993a,b) and the *DSCR1* gene, which is a regulator of calcineurin and in some circumstances, can inhibit calcineurin signaling pathways. It has been shown that calcineurin regulates Ca^2+^ pumps and exchangers to maintain Ca^2+^ homeostasis (Stark, 1996). Having an extra copy of *DSCR1* could potentially change calcium homeostasis in the brains of people with DS by influencing the activity of calcineurin, leading to formation of neurofibrillary tangles and consequently memory loss.

In AD, Aβ binds to neurons in close proximity to the NMDA-R which triggers NMDA-R- mediated calcium influx and alters calcium homeostasis (De Felice et al., 2007). Another report suggesting altered calcium homeostasis contributed to AD came from a study on L-type voltage gated calcium channels (LTVGCC) (Anekonda et al., 2011). In this study MC65 neuroblastoma cells were exogenously transfected with APP under the control of a tetracycline-responsive promoter. Upon the withdrawal of tetracycline, production of APP and Aβ fragments was observed along with up-regulation of LTVGCC, leading to increased calcium influx (Anekonda et al., 2011). Interestingly the use of calcium channel blockers prevented the neurotoxicity of Aβ. It has been suggested that the neurotoxicity of Aβ is due to the presence of elevated calcium ions, resulting in increased responses to excitatory amino acids such as glutamate (Mattson et al., 1993a). Due to these increased responses, glutamate receptors are over activated which result in dendritic pruning, increased immunoreactivity of tau, and an accumulation of filaments. These are all characteristics of neurofibrillary tangles of AD in DS and non-DS individuals.

## Conclusion

An extra dosage of the genes on chromosome 21 could contribute directly or indirectly (by changing metal homeostasis) to the manifestation of AD-like neuropathology in DS. There might be more genes involved in these processes other than the ones discussed in this review. Although some studies have been conducted on cognitive decline in DS individuals, the molecular mechanisms involved in this cognitive decline remain unknown. More studies are required to investigate these mechanisms and thus provide avenues to explore for the prevention of the cognitive decline characteristic of AD in DS and in the non-DS population.

## Conflict of interest statement

The authors declare that the research was conducted in the absence of any commercial or financial relationships that could be construed as a potential conflict of interest.

## References

[B1] Acevedo K. M. Opazo C. M. Norrish D. Challis L. M. Li Q.-X. White A. R. (2014). Phosphorylation of amyloid precursor protein at threonine-668 is essential for its copper-responsive trafficking in SH-SY5Y neuroblastoma cells. J. Biol. Chem. 289, 11007–11019 10.1074/jbc.m113.538710 24610780 PMC4036242

[B2] Adlard P. A. Bush A. I. (2006). Metals and Alzheimer’s disease. J. Alzheimers Dis. 10, 145–163 17119284 10.3233/jad-2006-102-303

[B3] Adlard P. A. Parncutt J. M. Finkelstein D. I. Bush A. I. (2010). Cognitive loss in zinc transporter-3 knock-out mice: a phenocopy for the synaptic and memory deficits of Alzheimer’s disease? J. Neurosci. 30, 1631–1636 10.1523/JNEUROSCI.5255-09.2010 20130173 PMC6633978

[B4] Alzheimer A. Stelzmann R. Schnitzlein H. N. Murtagh F. R. (1995). An English translation of Alzheimer’s 1907 paper, “Uber eine eigenartige Erkankung der Hirnrinde”. Clin. Anat. 8, 429–431 10.1002/ca.980080612 8713166

[B5] Anekonda T. S. Quinn J. F. Harris C. Frahler K. Wadsworth T. L. Woltjer R. L. (2011). L-type voltage-gated calcium channel blockade with isradipine as a therapeutic strategy for Alzheimer’s disease. Neurobiol. Dis. 41, 62–70 10.1016/j.nbd.2010.08.020 20816785 PMC2982927

[B6] Barnham K. J. Mckinstry W. J. Multhaup G. Galatis D. Morton C. J. Curtain C. C. (2003). Structure of the Alzheimer’s disease amyloid precursor protein copper binding domain a regulator of neuronal copper homeostasis. J. Biol. Chem. 278, 17401–17407 10.1074/jbc.m300629200 12611883

[B7] Bonda D. J. Lee H.-G. Blair J. A. Zhu X. Perry G. Smith M. A. (2011). Role of metal dyshomeostasis in Alzheimer’s disease. Metallomics 3, 267–270 10.1039/c0mt00074d 21298161 PMC3117398

[B8] Burch P. Milunsky A. (1969). Early-onset diabetes mellitus in the general and Down’s syndrome populations: genetics, aetiology and pathogenesis. Lancet 293, 554–558 10.1016/s0140-6736(69)91961-8 4179841

[B9] Bush A. I. (2013). The metal theory of Alzheimer’s disease. J. Alzheimers Dis. 33, S277–S281 10.3233/JAD-2012-129011 22635102

[B10] Bush A. I. Pettingell W. Paradis M. Tanzi R. E. (1994). Modulation of a beta adhesiveness and secretase site cleavage by zinc. J. Biol. Chem. 269, 12152–12158 8163520

[B11] Cataldo A. M. Petanceska S. Peterhoff C. M. Terio N. B. Epstein C. J. Villar A. (2003). App gene dosage modulates endosomal abnormalities of Alzheimer’s disease in a segmental trisomy 16 mouse model of down syndrome. J. Neurosci. 23, 6788–6792 12890772 10.1523/JNEUROSCI.23-17-06788.2003 PMC6740714

[B12] Cataldo A. M. Peterhoff C. M. Troncoso J. C. Gomez-Isla T. Hyman B. T. Nixon R. A. (2000). Endocytic pathway abnormalities precede amyloid β deposition in sporadic Alzheimer’s disease and down syndrome: differential effects of apoe genotype and presenilin mutations. Am. J. Pathol. 157, 277–286 10.1016/s0002-9440(10)64538-5 10880397 PMC1850219

[B13] Cater M. Mcinnes K. Li Q. Volitakis I. La Fontaine S. Mercer J. (2008). Intracellular copper deficiency increases amyloid-beta secretion by diverse mechanisms. Biochem. J. 412, 141–152 10.1042/BJ20080103 18248325

[B14] Cheon M. S. Dierssen M. Kim S. H. Lubec G. (2008). Protein expression of BACE1, BACE2 and APP in Down syndrome brains. Amino Acids 35, 339–343 10.1007/s00726-007-0618-9 18163181

[B15] Cole T. B. Wenzel H. J. Kafer K. E. Schwatzkorin P. A. Palmiter R. D. (1999). Elimination of zinc from synaptic vesicles in the intact mouse brain by disruption of the ZnT3 gene. Proc. Natl. Acad. Sci. U S A 96, 1716–1721 10.1073/pnas.96.4.1716 9990090 PMC15571

[B16] Contestabile A. Benfenati F. Gasparini L. (2010). Communication breaks-down: from neurodevelopment defects to cognitive disabilities in down syndrome. Prog. Neurobiol. 91, 1–22 10.1016/j.pneurobio.2010.01.003 20097253

[B17] Dahms S. O. Könnig I. Roeser D. Gührs K.-H. Mayer M. C. Kaden D. (2012). Metal binding dictates conformation and function of the amyloid precursor protein (APP) E2 domain. J. Mol. Biol. 416, 438–452 10.1016/j.jmb.2011.12.057 22245578

[B18] De Felice F. G. Velasco P. T. Lambert M. P. Viola K. Fernandez S. J. Ferreira S. T. (2007). Aβ oligomers induce neuronal oxidative stress through an N-methyl-D-aspartate receptor-dependent mechanism that is blocked by the Alzheimer drug memantine. J. Biol. Chem. 282, 11590–11601 10.1074/jbc.M607483200 17308309

[B19] Duce J. A. Tsatsanis A. Cater M. A. James S. A. Robb E. Wikhe K. (2010). Iron-export ferroxidase activity of β-amyloid precursor protein is inhibited by zinc in Alzheimer’s disease. Cell 142, 857–867 10.1016/j.cell.2010.08.014 20817278 PMC2943017

[B20] Epstein C. J. (1995). “The metabolic and molecular bases of inherited disease,” in Down Syndrome (Trisomy 21), eds C. Scriver, A. L. Beaudet, W. S. Sly and D. Vale (New York: McGraw-Hill, Inc.), 749–794

[B21] Ermak G. Morgan T. E. Davies K. J. (2001). Chronic overexpression of the calcineurin inhibitory gene DSCR1 (Adapt78) is associated with Alzheimer’s disease. J. Biol. Chem. 276, 38787–38794 10.1074/jbc.m102829200 11483593

[B22] Ermak G. Pritchard M. A. Dronjak S. Niu B. Davies K. J. (2011). Do RCAN1 proteins link chronic stress with neurodegeneration? FASEB J. 25, 3306–3311 10.1096/fj.11-185728 21680892 PMC3971512

[B23] Farrar G. Altmann P. Welch S. Wychrij O. Ghose B. Lejeune J. (1990). Defective gallium-transferrin binding in Alzheimer disease and down syndrome: possible mechanism for accumulation of aluminium in brain. Lancet 335, 747–750 10.1016/0140-6736(90)90868-6 1969510

[B24] Finefrock A. E. Bush A. I. Doraiswamy P. M. (2003). Current status of metals as therapeutic targets in Alzheimer’s disease. J. Am. Geriatr. Soc. 51, 1143–1148 10.1046/j.1532-5415.2003.51368.x 12890080

[B25] Frederickson C. J. Klitenick M. A. Manton W. I. Kirkpatrick J. B. (1983). Cytoarchitectonic distribution of zinc in the hippocampus of man and the rat. Brain Res. 273, 335–339 10.1016/0006-8993(83)90858-2 6616240

[B64] Frederickson C. J. Suh S. W. Silva D. Frederickson C. J. Thompson R. B. (2000). Importance of zinc in the central nervous system: the zinc-containing neuron. J. Nutr. 130, 1471S–1483S 10801962 10.1093/jn/130.5.1471S

[B26] Fuentes J. J. Genesca L. Kingsbury T. J. Cunningham K. W. Perez-Riba M. Estivill X. (2000). DSCR1, overexpressed in down syndrome, is an inhibitor of calcineurin-mediated signaling pathways. Hum. Mol. Genet. 9, 1681–1690 10.1093/hmg/9.11.1681 10861295

[B27] Galdzicki Z. Coan E. Rapoport S. Stoll J. (1998). Increased expression of voltage-activated calcium channels in cultured hippocampal neurons from mouse trisomy 16, a model for Down syndrome. Brain Res. Mol. Brain Res. 56, 200–206 10.1016/s0169-328x(98)00046-1 9602127

[B28] Gerlach M. Ben-Shachar D. Riederer P. Youdim M. (1994). Altered brain metabolism of iron as a cause of neurodegenerative diseases? J. Neurochem. 63, 793–807 10.1046/j.1471-4159.1994.63030793.x 7519659

[B29] Greenberg S. Bacskai B. Hyman B. (2003). Alzheimer disease’s double-edged vaccine. Nat. Med. 9, 389–390 10.1038/nm847 12640449

[B30] Hattori M. Fujiyama A. Taylor T. Watanabe H. Yada T. Park H.-S. (2000). The DNA sequence of human chromosome 21. Nature 405, 311–319 10.1038/35012518 10830953

[B31] Huang X. Atwood C. S. Hartshorn M. A. Multhaup G. Scarpa R. C. Cuajungco M. P. (1999). The Aβ peptide of Alzheimer’s disease directlyproduces hydrogen peroxide through metal ion reduction. Biochemistry 38, 7609–7616 10.1021/bi990438f 10386999

[B32] Hyman B. T. Van Hoesen G. W. Kromer L. J. Damasio A. R. (1986). Perforant pathway changes and the memory impairment of Alzheimer’s disease. Ann. Neurol. 20, 472–481 10.1002/ana.410200406 3789663

[B33] Ikeda S. Yanagisawa N. Allsop D. Glenner G. G. (1989). Evidence of amyloid beta-protein immunoreactive early plaque lesions in Down’s syndrome brains. Lab. Invest. 61, 133–137 2473275

[B34] Jang H. Arce F. T. Ramachandran S. Capone R. Azimova R. Kagan B. L. (2010). Truncated beta-amyloid peptide channels provide an alternative mechanism for Alzheimer’s disease and down syndrome. Proc. Natl. Acad. Sci. U S A 107, 6538–6543 10.1073/pnas.0914251107 20308552 PMC2851998

[B35] Kardos J. Kovács I. Hajós F. Kálmán M. Simonyi M. (1989). Nerve endings from rat brain tissue release copper upon depolarization. A possible role in regulating neuronal excitability. Neurosci. Lett. 103, 139–144 10.1016/0304-3940(89)90565-x 2549468

[B36] Klomp L. Farhangrazi Z. S. Dugan L. L. Gitlin J. D. (1996). Ceruloplasmin gene expression in the murine central nervous system. J. Clin. Invest. 98, 207–215 10.1172/jci118768 8690795 PMC507418

[B37] Lei P. Ayton S. Finkelstein D. I. Spoerri L. Ciccotosto G. D. Wright D. K. (2012). Tau deficiency induces parkinsonism with dementia by impairing APP-mediated iron export. Nat. Med. 18, 291–295 10.1038/nm.2613 22286308

[B38] Lemere C. Blusztajn J. Yamaguchi H. Wisniewski T. Saido T. Selkoe D. (1996). Sequence of deposition of heterogeneous amyloid β-peptides and APO E in Down syndrome: implications for initial events in amyloid plaque formation. Neurobiol. Dis. 3, 16–32 10.1006/nbdi.1996.0003 9173910

[B39] Leveugle B. Spik G. Perl D. P. Bouras C. Fillit H. M. Hof P. R. (1994). The iron-binding protein lactotransferrin is present in pathologic lesions in a variety of neurodegenerative disorders: a comparative immunohistochemical analysis. Brain Res. 650, 20–31 10.1016/0006-8993(94)90202-x 7953673

[B40] Loeffler D. Connor J. Juneau P. Snyder B. Kanaley L. Demaggio A. (1995). Transferrin and iron in normal, Alzheimer’s disease and Parkinson’s disease brain regions. J. Neurochem. 65, 710–716 10.1046/j.1471-4159.1995.65020710.x 7616227

[B41] Lyubartseva G. Smith J. L. Markesbery W. R. Lovell M. A. (2010). Alterations of zinc transporter proteins ZnT-1, ZnT-4 and ZnT-6 in preclinical Alzheimer’s disease brain. Brain Pathol. 20, 343–350 10.1111/j.1750-3639.2009.00283.x 19371353 PMC3175637

[B42] Ma H. Xiong H. Liu T. Zhang L. Godzik A. Zhang Z. (2004). Aggregate formation and synaptic abnormality induced by DSCR1. J. Neurochem. 88, 1485–1496 10.1046/j.1471-4159.2003.02294.x 15009650

[B43] Mann D. (1988). The pathological association between Down syndrome and Alzheimer disease. Mech. Ageing Dev. 43, 99–136 10.1016/0047-6374(88)90041-3 2969441

[B44] Mann D. M. Jones D. Prinja D. Purkiss M. S. (1990). The prevalance of amyloid (A4) protein depositis within the cereblar and cerebellar cortex in Down’s syndrome and Alzheimer’s dosease. Acta Neuropathol. 80, 318–327 10.1007/bf00294651 1698007

[B45] Mattson M. P. Barger S. W. Cheng B. Lieberburg I. Smith-Swintosky V. L. Rydel R. E. (1993a). beta-Amyloid precursor protein metabolites and loss of neuronal Ca2+ homeostasis in Alzheimer’s disease. Trends Neurosci. 16, 409–414 10.1016/0166-2236(93)90009-b 7504356

[B46] Mattson M. P. Tomaselli K. J. Rydel R. E. (1993b). Calcium-destabilizing and neurodegenerative effects of aggregated β-amyloid peptide are attenuated by basic FGF. Brain Res. 621, 35–49 10.1016/0006-8993(93)90295-x 8221072

[B47] Maynard C. J. Cappai R. Volitakis I. Cherny R. A. White A. R. Beyreuther K. (2002). Overexpression of Alzheimer’s disease amyloid-β opposes the age-dependent elevations of brain copper and iron. J. Biol. Chem. 277, 44670–44676 10.1074/jbc.m204379200 12215434

[B48] Mok K. Y. Jones E. L. Hanney M. Harold D. Sims R. Williams J. (2014). Polymorphisms in *BACE2* may affect the age of onset Alzheimer’s dementia in Down syndrome. Neurobiol. Aging 35, 1513.e1–1513.e5 10.1016/j.neurobiolaging.2013.12.022 24462566 PMC3969241

[B49] Nalbandyan R. (1983). Copper in brain. Neurochem. Res. 8, 1211–1232 10.1007/bf00963993 6419137

[B50] Peiris H. Raghupathi R. Jessup C. F. Zanin M. P. Mohanasundaram D. Mackenzie K. D. (2012). Increased expression of the glucose-responsive gene, RCAN1, causes hypoinsulinemia, β-cell dysfunction and diabetes. Endocrinology 153, 5212–5221 10.1210/en.2011-2149 23011918

[B51] Perluigi M. di Domenico F. Fiorini A. Cocciolo A. Giorgi A. Foppoli C. (2011). Oxidative stress occurs early in Down syndrome pregnancy: a redox proteomics analysis of amniotic fluid. Proteomics Clin. Appl. 5, 167–178 10.1002/prca.201000121 21360684

[B52] Pike C. J. Overman M. J. Cotman C. W. (1995). Amino-terminal deletions enhance aggregation of β-amyloid peptides in vitro. J. Biol. Chem. 270, 23895–23898 10.1074/jbc.270.41.23895 7592576

[B53] Piñero D. J. Connor J. R. (2000). Iron in the brain: an important contributor in normal and diseased states. Neuroscientist 6, 435–453 10.1177/107385840000600607

[B54] Prasher V. P. Farrer M. J. Kessling A. M. Fisher E. M. C. West R. J. Barber P. C. (1998). Molecular mapping of alzheimer-type dementia in Down’s syndrome. Ann. Neurol. 43, 380–383 10.1002/ana.410430316 9506555

[B55] Sayre L. M. Perry G. Harris P. L. Liu Y. Schubert K. A. Smith M. A. (2000). In situ oxidative catalysis by neurofibrillary tangles and senile plaques in Alzheimer’s disease. J. Neurochem. 74, 270–279 10.1046/j.1471-4159.2000.0740270.x 10617129

[B56] Schupf N. Sergievsky G. H. (2002). Genetic and host factors for dementia in Down’s syndrome. Br. J. Psychiatry 180, 405–410 10.1192/bjp.180.5.405 11983636

[B57] Sensi S. L. Paoletti P. Koh J.-Y. Aizenman E. Bush A. I. Hershfinkel M. (2011). The neurophysiology and pathology of brain zinc. J. Neurosci. 31, 16076–16085 10.1523/jneurosci.3454-11.2011 22072659 PMC3223736

[B58] Sindreu C. Storm D. R. (2011). Modulation of neuronal signal transduction and memory formation by synaptic zinc. Front. Behav. Neurosci. 5:68 10.3389/fnbeh.2011.00068 22084630 PMC3211062

[B59] Smith M. A. Wehr K. Harris P. L. Siedlak S. L. Connor J. R. Perry G. (1998). Abnormal localization of iron regulatory protein in Alzheimer’s disease. Brain Res. 788, 232–236 10.1016/s0006-8993(98)00002-x 9555030

[B60] Stark M. J. R. (1996). Yeast protein serine/threonine phosphatases: multiple roles and diverse regulation. Yeast 12, 1647–1675 10.1002/(SICI)1097-0061(199612)12:16<1647::AID-YEA71>3.0.CO;2-Q 9123967

[B61] Vassar R. Bennett B. D. Babu-Khan S. Kahn S. Mendiaz E. A. Denis P. (1999). [Beta]-secretase cleavage of Alzheimer’s amyloid precursor protein by the transmembrane aspartic protease BACE. Science 286, 735–741 10.1126/science.286.5440.735 10531052

[B62] White A. R. Reyes R. Mercer J. F. Camakaris J. Zheng H. Bush A. I. (1999). Copper levels are increased in the cerebral cortex and liver of APP and APLP2 knockout mice. Brain Res. 842, 439–444 10.1016/s0006-8993(99)01861-2 10526140

[B63] Wilcock D. M. Griffin W. S. (2013). Down’s syndrome, neuroinflammation and Alzheimer neuropathogenesis. J. Neuroinflammation 10:84 10.1186/1742-2094-10-84 23866266 PMC3750399

